# Elevated level of circulating VEGF in Chinese patients with hereditary angioedema and its correlation with disease status

**DOI:** 10.1186/s13023-025-03776-3

**Published:** 2025-05-26

**Authors:** Ruoyu Ji, Yijing Xu, Yuxiang Zhi

**Affiliations:** Department of Allergy, Peking Union Medical College, Peking Union Medical College Hospital, Chinese Academy of Medical Sciences, Beijing, 100730 China

**Keywords:** Hereditary angioedema, Vascular endothelium, VEGF, Disease severity, Biomarker

## Abstract

**Background:**

Hereditary angioedema (HAE) is a rare inherited disease characterized by recurrent, potentially life-threatening angioedema. The vascular endothelium dysfunction is reported to play a role in angioedema episodes. Here, we conducted a case-control study to explore the correlation between vascular endothelium growth factor (VEGF), a representative indicator for endothelium dysfunction, and HAE as well as its attack frequency, disease control and disease severity.

**Methods:**

Patients with HAE and non-hereditary angioedema in their attack-free period were prospectively recruited. Demographic and disease information were collected through questionnaires. Disease control of HAE was assessed with the angioedema control test (AECT) with a recall period of three months. The current severity of HAE was comprehensively assessed through frequency of angioedema episodes, occurrence of life-threatening angioedema, necessity for hospitalization or emergency department visits. The plasma VEGF level was measured by chemiluminescence microparticle immunoassay. We compared clinical characteristics between HAE and non-hereditary angioedema patients, as well as among HAE patients with different attack frequency, disease control and disease severity. We further performed several generalized linear models (GLMs) to examine the correlation between VEGF levels and the attack frequency, disease control and disease severity of HAE.

**Results:**

We enrolled 74 patients with HAE and 55 patients with non-hereditary angioedema. HAE patients exhibited higher VEGF levels in remission than controls (112 vs. 60 ng/ml, *P* < 0.001). VEGF levels further increased in HAE patients with more frequent angioedema attacks, poorer disease control and greater disease severity. Results of GLMs confirmed significant correlations between plasma VEGF concentrations and the attack frequency of angioedema, disease control status and disease severity of HAE.

**Conclusion:**

Circulating VEGF level elevated in patients with HAE during attack-free periods, particularly among those with greater disease burden, suggesting the involvement of vascular endothelial dysfunction in the pathogenesis of HAE. VEGF may serve as a predictive biomarker for risk stratification and disease monitoring in HAE.

**Supplementary Information:**

The online version contains supplementary material available at 10.1186/s13023-025-03776-3.

## Introduction

Hereditary angioedema (HAE) is a rare inherited disorder characterized by recurrent subcutaneous or mucosal angioedema, including bowel wall edema and laryngeal edema [1]. Angioedema attacks are mostly unpredictable and could be life-threatening, imposing considerable physical and psychological burdens on patients [2, 3]. In the vast majority of cases, HAE is caused by the causative mutations in complement 1 (C1) inhibitor gene (SERPING1) that leads to C1-esterase inhibitor (C1-INH) deficiency (type I) or dysfunction (type II), resulting in uncontrolled activation of kallikrein–kinin system and overproduction of bradykinin [4, 5]. On this basis, current therapeutic strategies aim to aim to inhibit specific targets within the kallikrein–kinin system (e.g., lanadelumab, berotralstat, and ecallantide inhibit plasma kallikrein), its upstream pathways (e.g., garadacimab inhibits Factor XII), or its downstream pathways (e.g., icatibant inhibits bradykinin receptor) [6, 7]. However, disease activity and therapeutic efficacy among HAE patients are very heterogeneous clinically, which inspires further search for more biomarkers in various pathophysiologic pathways [8]. Results of previous studies indicated that levels of various factors related to increased vascular permeability, including vascular endothelial growth factor (VEGF), were higher in HAE patients compared to controls even at the remission state, and were further elevated in HAE patients with more frequent episodes, suggesting a predisposition to high vascular permeability in HAE pathogenesis [9, 10]. VEGF, the key biomarker of endothelial function [11], has recently been found as a major hub gene in top 2 enriched gene networks revealed by a blood transcriptome analysis based on HAE patients with prodromal symptoms [12]. The predictive ability of VEGF in HAE, as well as its crosstalk with the bradykinin pathway has been largely unrevealed. Here, we compared circulating VEGF levels in Chinese HAE and non-hereditary angioedema patients and further explored its relationship with frequency of angioedema attack, disease control status and disease severity of HAE and its possible role in pathogenic mechanisms.

## Methods and materials

This case-control study complied with the Declaration of Helsinki and was approved by the Ethics Committee of Peking Union Medical College Hospital (ethics approval number: I-24PJ2095). Informed consents were obtained from all patients. This article adheres to the Strengthening the Reporting of Observational Studies in Epidemiology-Molecular Epidemiology (STROBE-ME) guidelines [13], with the checklist demonstrated in Table S1.

### Participants

We prospectively enrolled recurrent hereditary and non-hereditary angioedema patients in an attack-free period at our specialist angioedema outpatient clinic from January 2024 to November 30, 2024. The diagnosis of HAE type I/type II was made based on the following criteria: (1) documented clinical manifestations of recurrent angioedema (2) C1-INH level < 50% of the lower normal range for type I HAE, normal or slightly increased C1-INH level with functional activity < 50% of the lower normal range for type II HAE. (3) a decreased complement 4 (C4) antigen concentration below the lower limit of the reference range, and a normal complement 1q (C1q) level; (4) a definite family history of angioedema or a pathogenic mutation in genes [14]. The non-hereditary angioedema was diagnosed according to the DANCE consensus, which was defined as a paroxysmal, localized, and self-limiting swelling of the subcutaneous and/or submucosal tissue [15]. Patients having acquired angioedema with a decreased C1-IHN level was not included due to its overlapping pathogenesis with HAE. The inclusion criteria for both groups were: (1) aged ≥ 12 years; (2) capable of providing written informed consent; (3) in the attack-free period. Exclusion criteria were established to eliminate the influence of factors known to directly affect vascular permeability. The exclusion criteria for both groups were: (1) use of antihistamines, glucocorticoid or immunosuppressant or other drugs that were known to directly affect vascular permeability within five half-lives of the corresponding drug before collection of blood sample; (2) concomitant diagnosis of severe systemic disorders; (3) infection during the last two weeks; (4) pregnancy or lactation.

### Data collection and processing

Enrolled patients were asked to fill in the questionnaire under the instruction of researchers. The questionnaire collected the following information: baseline demographic characteristics, family history, age of symptom onset, age of diagnosis and the site, duration, accompanied symptoms as well as treatment response of angioedema (including abdominal and laryngeal edema). For HAE patients, we additionally collected objective indicators including the number of angioedema attacks, the presence of laryngeal edema, the presence of intense abdominal pain, long-term prophylaxis (LTP), outpatient and emergency visit and treatments, as well as subjective information including the impact on quality of life, the degree of unpredictability and self-assessed disease control status during the past three and six months.

#### Disease control of HAE

We utilized the angioedema control test (AECT) scale with a recall period of 3 months to assess the disease control status of HAE [16]. The total AECT score was determined by aggregating the scores of four items: attack frequency, impact on quality of life, unpredictability of angioedema, and self-assessed disease control through medication, utilizing the information collected in the questionnaire. In cases where patients did not receive LTP, imputation was conducted by substituting the rounded mean score of the available three items. The score ranged from a minimum of 0 to a maximum of 16 points. The higher the AECT score the better the control of HAE, and a cut-off value of 10 points to set distinguish patients with poorly controlled and well-controlled HAE [14].

#### Disease severity of HAE

Evaluation criteria for current disease severity of HAE was modified from a published severity score [17] and the criteria proposed by our institution comprehensively [18]. Patients who met either of the following criteria were categorized into the severe disease group: 1) ≥ 6 angioedema attacks in the past six months; 2) any life-threatening angioedema in the past six months. Life-threatening angioedema was defined as laryngeal angioedema or recurrent, intense abdominal angioedema requiring hospitalization or emergency visit. In this study, the receipt of LTP was not included as a criterion for determining severe disease status, as the primary focus was on symptomatic manifestations. The disease severity was evaluated independently by two researchers with disagreements solved through group discussion.

### Circulating VEGF level

#### Sample collection, Preparation and storage

Patients were required to stop taking antihistamines for at least three days prior to blood test. For patients receiving lanadelumab, blood samples were collected before the next injection. Blood samples were obtained concurrently with routine diagnostic procedures by experienced nurses. The samples were collected in Vacutainer© tubes (BD, USA) and mixed at a ratio of 1:9 with 3.2% sodium citrate. Following centrifugation at 3000 rpm for 10 min at 4 °C, the supernatant plasma was isolated and stored at − 80 °C before testing.

#### Measurement of Circulating VEGF level

The plasma VEGF level was measured utilizing the chemiluminescence microparticle immunoassay on Aurora 2000i (Kanghua Biotechnology Co., Ltd, Shandong, China) according to the manufacturer’s instructions. The equipment was calibrated before every test. The normal range of VEGF level is < 160 ng/ml in our institution.

### Statistical analyses

Continuous variables were described as median (interquartile range (IQR), [p25–p75]) or mean ± standard deviation (SD) according to whether the distribution of the data strictly followed the normal distribution. Categorical variables were described as absolute value and percentages. Comparisons of continuous variables between two groups were performed with Mann-Whitney U test. Comparisons of continuous variables among multiple groups were made by Kruskal-Wallis test, with between-group data further compared using Kruskal-Wallis ANOVA test and adjusted P values were calculated by the Bonferroni correction. Categorical variables were compared by chi-square test or Fisher exact test, as appropriate. Additionally, a receiver operating characteristic (ROC) analysis was conducted to evaluate the predictive value of VEGF for HAE, as determined by the area under curve (AUC).

To assess the association between VEGF levels and attack frequency over the previous six months, multiple generalized linear models (GLMs) were developed and compared. Given that the proportion of zero values (17.6%) was significantly higher than those predicted by the standard Poisson model (3.1%) and the standard negative binomial model (4.4%), zero-inflated models were employed. The optimal main effect model was selected based on the lowest Akaike Information Criterion (AIC) values. Results were presented as incidence rate ratios (IRR), 95% confidence intervals (CI), and P-values. Model fit was evaluated using McFadden’s Pseudo R-squared (McFadden R²) [19], and the Vuong test was utilized to confirm the superiority of zero-inflated models over the standard Poisson or negative binomial models [20].

In examining the association between circulating VEGF levels and AECT scores, a bivariate Pearson’s correlation analysis was conducted, followed by a multivariate linear regression analyses. The main-effect model characterized by the smallest values of AIC value was selected as the optimal model. Results are reported as beta-coefficients (B), standard errors (SE) and P values. We employed the root mean square error (RMSE) and coefficient of determination (R^2^) to test model fitness, and we utilized variance inflation factors (VIF) to evaluate the presence of multicollinearity among the variables within the model.

To evaluate the correlation of VEGF level and disease severity and to explore potential risk factors for the severe disease status of HAE, we employed the univariate and multivariate logistic regression analysis. Clinical and laboratory indexes with P value < 0.05 in the univariate analysis and baseline demographic characteristics were subsequently included in the multivariate regression model to identify independent risk factors for severe HAE. Results are reported as odds ratios (OR), 95% CIs and P values.

Data analyses were performed using SPSS 26.0 for windows (SPSS institute, Chicago, IL, USA) and the R software (Version 4.2.3). Sample size estimation and post-hoc power calculation were conducted using the G*Power software (Version 3.1.9.7, Germany) with results detailed in the Supplementary Material.

## Results

### Patient selection

**Fig. 1:**
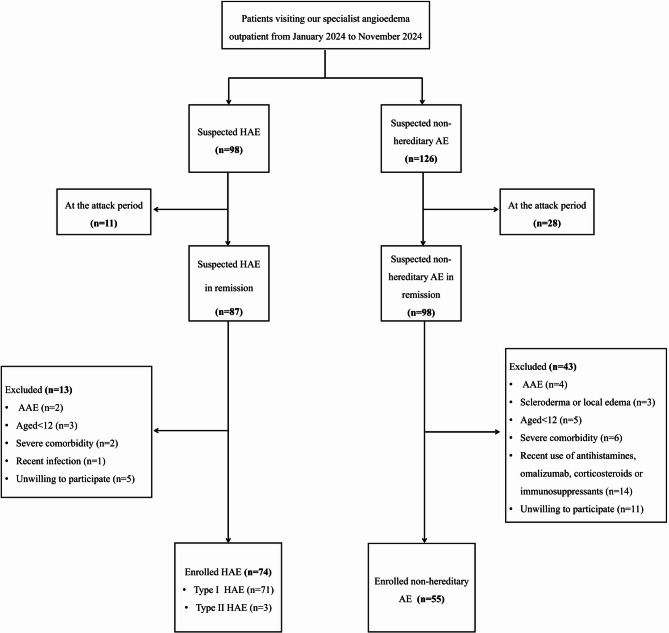
Flow diagram for patient enrollment. HAE, hereditary angioedema; AE, angioedema; AAE, acquired angioedema

A total of 74 patients with HAE and 55 patients with non-hereditary angioedema satisfied the inclusion and exclusion criteria and were subsequently enrolled in the study. The enrollment process was illustrated in Fig. 1. Within the cohort of HAE patients, three individuals (4.1%) were diagnosed with type II HAE. At the time of enrollment, 25 patients (33.8%) were undergoing LTP, with 20 receiving danazol and 5 receiving lanadelumab.

### Clinical comparisons of HAE and non-hereditary angioedema patients

**Fig. 2:**
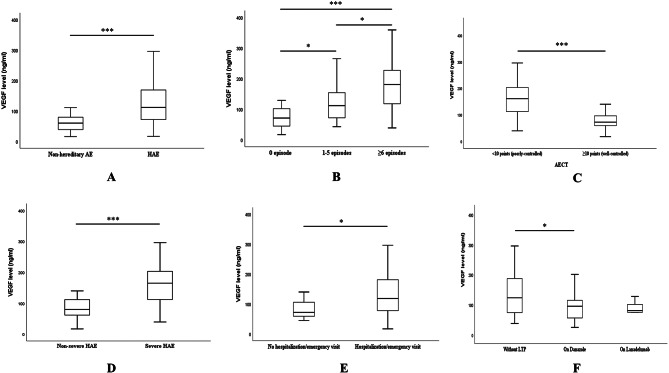
Comparisons of circulating VEGF levels between HAE and non-hereditary angioedema (**A**), HAE with different attack frequency (**B**), HAE with different disease control status by AECT scores (**C**), HAE with different disease severity (**D**), HAE with or without a history of hospitalization or emergency visit (**E**), HAE with LTP (danazol or lanadelumab) or without LTP (**F**). VEGF, vascular endothelium growth factor; HAE, hereditary angioedema; AECT, Angioedema control test; LTP, long-term prophylaxis. ^***^P < 0.001, ^**^P < 0.01, ^*^P < 0.05

The demographic and disease characteristics were tabulated in Table 1. The age, sex and body mass index were comparable between groups. Patients with non-hereditary angioedema were more likely to comorbid other atopic diseases (43.6% vs. 8.1%, *P* < 0.001). Compared to the control group, patients with HAE had earlier age of onset (24.0 [16.8–29.3] vs. 39.0 [27.5–49.7] years, *P* < 0.001), longer disease course (18.0 [8.0–24.0] vs. 2.0 [0.5-5.0] years, *P* < 0.001) and higher proportion of a positive family history (85.1% vs. 9.1%, *P* < 0.001). HAE patients were more prone to experience laryngeal edema (60.8% vs. 25.5%, *P* < 0.001), intense abdominal pain (68.9% vs. 16.4%, *P* < 0.001) and hospitalization or emergency department visit (83.8% vs. 27.3%, *P* < 0.001), but were less likely to develop concurrent urticaria (0.0% vs. 23.6%, *P* < 0.001) during the angioedema attack throughout the disease course. Results of laboratory tests demonstrated that the HAE group had a higher level of VEGF (112 [72–169] vs. 60 [39–80] ng/ml, *P* < 0.001, Fig. 2A) but lower levels of C1-INH (C1-INH/lower normal limit, 0.24 [0.19–0.29] vs. 1.33 [1.19–1.52], *P* < 0.001) and C4 (0.053 [0.023–0.085] vs. 0.215 [0.178–0.269] mg/ml, *P* < 0.001). Further bivariate ROC analysis (Figure S1) with an AUC value of 0.823 confirmed the predictive capability of circulating VEGF level for the diagnosis of HAE.

**Table 1:** Baseline demographic and disease characteristics of enrolled patients

	HAE (*n* = 74)	Non-hereditary AE (*n* = 55)	*P* value
**Demographic characteristics**			
Age (years, median, IQR)	41(33–54)	43 (32–55)	0.954
Male (n, %)	30 (40.5%)	28 (50.9%)	0.762
BMI (kg/m^2^, median, IQR)	21.7 (19.4–23.8)	21.1 (19.6–23.0)	0.675
Non-allergic comorbidities (n, %)	24 (32.4%)	22 (40.0%)	0.375
Allergic comorbidity (n, %)	6 (8.1%)	24 (43.6%)	< 0.001
**Disease characteristics**			
Family history (n, %)	63 (85.1%)	5 (9.1%)	< 0.001
Age of onset (years, median, IQR)	24.0 (16.8–29.3)	39.0 (27.5–49.7)	< 0.001
Disease duration (years, median, IQR)	18.0 (8.0–24.0)	2.0 (0.5-5.0)	< 0.001
Hospitalization or emergency department visit (n, %)	62 (83.8%)	15 (27.3%)	< 0.001
History of laryngeal edema (n, %)	45 (60.8%)	14 (25.5%)	< 0.001
History of intense abdominal pain (n, %)	51 (68.9%)	9 (16.4%)	< 0.001
Concurrent urticaria (n, %)	0 (0.0%)	13 (23.6%)	< 0.001
**Drug interventions** (n, %)			
Danazol	20 (27.0%)	0 (0.0%)	< 0.001
Lanadelumab	5 (6.8%)	0 (0.0%)	
Second-generation antihistamines	0 (0.0%)	24 (43.6%)	
Glucocorticoid	0 (0.0%)	2 (3.6%)	
**Laboratory results**			
C1-INH/lower normal limit (median, IQR)	0.24 (0.19–0.29)	1.33 (1.19–1.52)	< 0.001
Complement 4 (mg/mL, median, IQR)	0.053 (0.023–0.085)	0.215 (0.178–0.269)	< 0.001
VEGF (ng/ml, median, IQR)	112 (72–169)	60 (39–80)	< 0.001

HAE, hereditary angioedema; AE, angioedema; IQR, interquartile range; BMI, body mass index; C1-INH, complement 1-esterase inhibitor; VEGF, vascular endothelium growth factor; AECA, anti-endothelial cell antibody

### Circulating VEGF level and attack frequency of angioedema

**Fig. 3:**
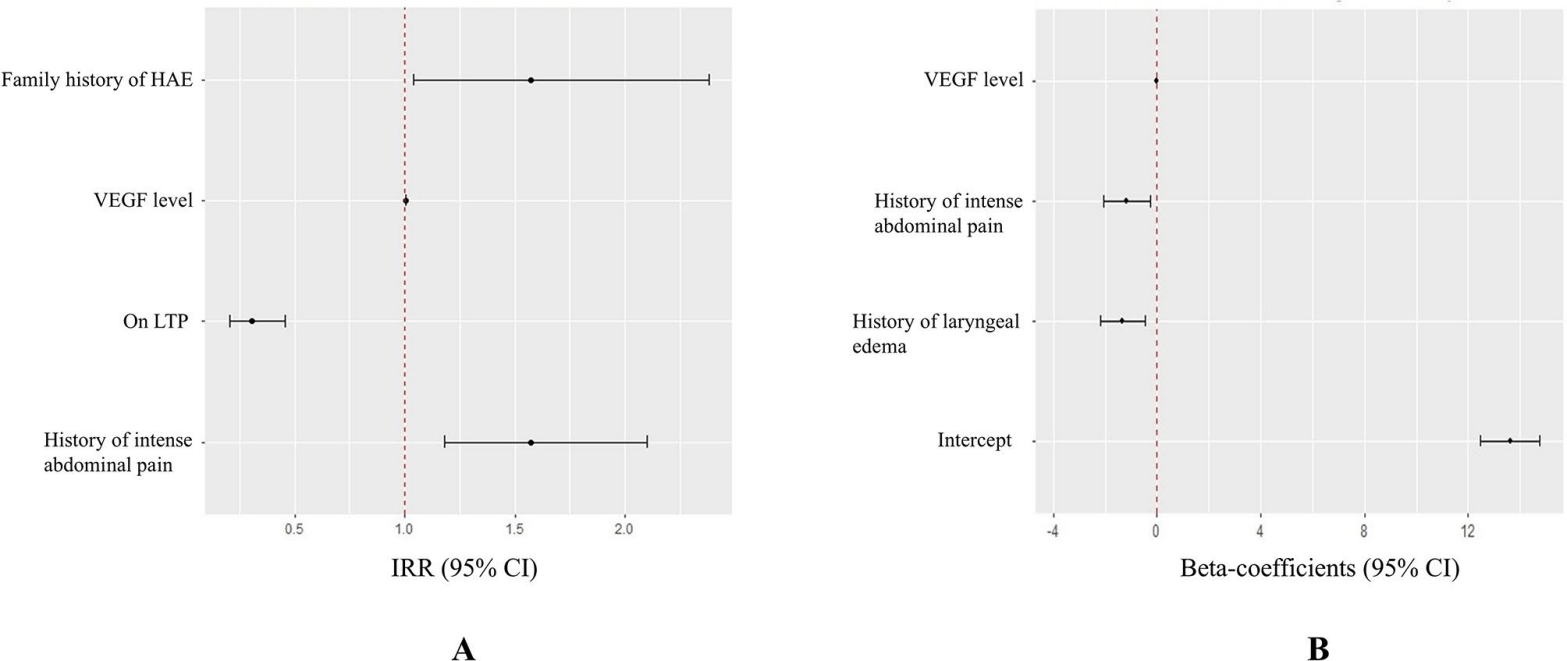
Forrest plots of the zero-inflated Poisson model (**A**) and the multivariate linear regression model (**B**). The zero-inflated Poisson model assessed the correlation between variables and number of angioedema attacks in the last six months. The multivariate linear regression model assessed the correlation between variables and AECT scores with a recall period of 3 months. VEGF, vascular endothelium growth factor; HAE, hereditary angioedema; AECT, angioedema control test; LTP, long-term prophylaxis; IRR, incidence rate ratios; CI, confidence interval

Among HAE patients, 13 were free of angioedema attacks in the past six months. The median number of angioedema attacks was 3 (IQR, 1–6). Patients who were attack-free exhibited significantly lower VEGF levels compared to those experiencing six or more attacks in the past six months (72 [43–103] vs. 181 [118–233] ng/ml, adjusted *P* < 0.001, Fig. 2B), as well as compared to those with less frequent attacks (72 [43–103] vs. 112 [72–157] ng/ml, adjusted *P* = 0.013, Fig. 2B). A significant difference was also observed among patients with varying frequencies of angioedema attacks (adjusted *P* = 0.029). The zero-inflated Poisson model exhibiting the lowest AIC value was selected as the optimal model for examining the correlation between VEGF levels and attack frequency of HAE (Fig. 3A). The Vuong test confirmed its superiority over the standard Poisson (*P* < 0.001) or negative binomial (*P* < 0.001) models. McFadden R² of this model was 0.230 which indicated good model fit (> 0.20). Results of this model indicated that higher VEGF level (IRR = 1.00, 95% CI 1.00 to 1.01, *P* < 0.001), history of intense abdominal pain (IRR = 1.57, 95% CI 1.18 to 2.10, *P* = 0.002) and family history of HAE (IRR = 1.57, 95% CI 1.04 to 2.38, *P* = 0.033) were associated with more frequent angioedema attacks, while receiving LTP was contributed to less frequent angioedema attacks (IRR = 0.30, 95% CI 0.20 to 0.46, *P* < 0.001).

### Circulating VEGF level and disease control status

The median AECT score was 9 (IQR, 5–11) among all HAE patients. 29 (39.2%) patients were categorized into the well-controlled subgroup. The VEGF level was significantly lower in well-controlled patients compared to poorly controlled patients (72 [56–99] vs. 161 [112–203] ng/ml, *P* < 0.001, Fig. 2C) The Pearson correlation analysis demonstrated a significant negative correlation between AECT scores and VEGF levels (*r*=-0.748, *P* < 0.001). Multivariate linear regression analysis (Fig. 3B) indicated that higher VEGF level (B=-0.03, SE = 0.01, *P* < 0.001), history of laryngeal edema (B=-1.32, SE = 0.44, *P* = 0.003) and history of intense abdominal pain (B=-1.17, SE = 0.45, *P* = 0.012) were associated with poor disease control. The R² and RMSE value of the model was 0.638 and 1.757 respectively, suggesting satisfied model fitness. VIF values of three variables were less than 1.1, suggesting that no multicollinearity among variables was detected.

### Circulating VEGF level and disease severity of HAE

According to our stratification criteria, 39 patients (52.7%) with HAE were classified as having severe disease, while 35 patients (47.3%) were categorized as having non-severe HAE. The clinical characteristics of these subgroups are presented in Table S2. Laboratory analyses revealed that patients with severe HAE exhibited elevated levels of VEGF (165 [112–203] ng/ml vs. 80 [61–112] ng/ml, *P* < 0.001, Fig. 2D) We conducted univariate and multivariate logistic regression analyses to identify risk factors associated with increased severity of HAE, as presented in Table 2. The univariate analysis revealed that a history of laryngeal edema (OR = 5.81, 95% CI 2.08 to 16.29, *P* = 0.001), lower C4 levels (OR = 0.25, 95% CI 0.07 to 0.84, *P* = 0.025), and elevated VEGF level (OR = 1.03, 95% CI 1.01 to 1.04, *P* < 0.001) were potential risk factors for more severe disease manifestations. In contrast, undergoing LTP demonstrated a protective effect (OR = 0.15, 95% CI 0.05 to 0.46, *P* = 0.001). The subsequent multivariate analysis confirmed that a history of laryngeal edema (OR = 25.04, 95% CI 3.62 to 173.45, *P* = 0.001) and elevated VEGF level (OR = 1.04, 95% CI 1.01 to 1.06, *P* = 0.001) were independent risk factors for increased disease severity, whereas receiving LTP was identified as an independent protective factor.

**Table 2:** Univariate and multivariate analyses of the correlation between clinical variables and the disease severity of HAE

Variables	Univariate analysis			Multivariate analysis		
	Odds ratio	95% CI	*P* value	Odds ratio	95% CI	*P* value
Age	1.00	0.97–1.03	0.887	1.02	0.97–1.07	0.420
Male	1.31	0.51–3.23	0.573	1.79	0.41–7.88	0.441
Comorbidity	0.85	0.32–2.26	0.747			
Duration of disease course	0.98	0.95–1.02	0.372	-	-	-
Family history	0.92	0.25–3.32	0.894	-	-	-
Previous laryngeal edema	5.81	2.08–16.29	0.001	25.04	3.62-173.45	0.001
Long-term prophylaxis	0.15	0.05–0.46	0.001	0.09	0.01–0.59	0.012
C1-INH/lower normal limit	0.23	0.030–1.74	0.153	-	-	-
Complement 4/lower normal limit	0.25	0.07–0.84	0.025	0.39	0.06–2.67	0.338
VEGF level	1.03	1.01–1.04	< 0.001	1.04	1.01–1.06	0.001

HAE, hereditary angioedema; CI, confidence interval; C1-INH, complement 1-esterase inhibitor; VEGF, vascular endothelium growth factor

### Comparison of VEGF level between other HAE subgroups

We additionally conducted several subgroup analyses to compare plasma VEGF levels among HAE patients with different clinical phenotypes. Additionally, VEGF levels were elevated in patients with a history of hospitalization or emergency visits due to angioedema compared to those without such history (119 [77–181] vs. 72 [57–108] ng/ml, *P* = 0.012, Fig. 2E). Patients treated with danazol for LTP exhibited significantly lower VEGF levels compared to those not receiving LTP (96 [55–117] ng/ml vs. 124 [74–190] ng/ml, adjusted *P* = 0.030, Fig. 2F). In contrast, no significant difference in VEGF levels was observed between patients receiving lanadelumab for LTP and those without LTP (82 [47–116] vs. 124 [74–190] ng/ml, adjusted *P* = 0.252), nor between patients treated with lanadelumab and those treated with danazol (adjusted *P* = 0.784).

## Discussion

The vascular endothelium functions as both an initiating and responsive element in the angioedema attacks of HAE [21]. To investigate the involvement of baseline vascular permeability in the pathogenesis of HAE and to explore its predictive role in disease monitoring, we conducted a comparative analysis of plasma levels of VEGF, a key regulator of vascular permeability, between patients with HAE and those with non-hereditary angioedema. Additionally, we compared VEGF levels among HAE patients with varying attack frequency, disease control status and disease severity during the attack-free period. Our findings demonstrated that VEGF levels were significantly elevated in HAE patients compared to those with non-hereditary angioedema, even during attack-free intervals. Subgroup analysis further demonstrated that VEGF levels were markedly elevated in HAE patients with more frequent attacks, poorer disease control and greater disease severity compared to those with less frequent and severe manifestations, suggesting the potential role of circulating VEGF as a biomarker for disease monitoring.

The heterogeneity in clinical manifestations and the unpredictability of angioedema attacks in hereditary angioedema (HAE) have long posed challenges for both clinicians and patients. From a mechanistic perspective, the reasons for the localized and episodic nature of angioedema episodes remain unclear. Although it is widely recognized that the fundamental abnormality in HAE is the uncontrolled activation of the plasma contact system, primarily due to deficient functional C1-INH [1], there is no conclusive evidence that the concentration or functionality of C1-INH is significantly correlated with disease severity or activity, as demonstrated by our previous analyses of Chinese HAE patients [21, 22, 23]. Furthermore, SERPING1-deficient mice exhibit constitutive swelling rather than the paroxysmal angioedema observed in clinical settings [24]. In recent years, a growing body of research has highlighted the role of vascular endothelium in HAE [25, 26]. The updated classification of angioedema has introduced a distinct category termed “angioedema due to intrinsic vascular endothelium dysfunction,” which includes several rare subtypes of HAE and systemic capillary leak syndrome, suggesting an important and unique role for endothelial dysfunction in the pathogenesis of HAE [15]. Therefore, we hypothesized that a balance exists between the plasma contact system and the stability of the vascular endothelium during the attack-free period in patients with HAE. Disruption of this balance, whether due to excessive production of bradykinin or dysfunction of the vascular endothelium, can lead to the occurrence of angioedema. Our study found that the VEGF levels in HAE patients during attack-free periods were higher than those in controls with non-hereditary angioedema, corroborating findings from previous studies [27, 28, 29] that suggest persistent endothelial dysfunction in HAE patients. Furthermore, the vascular endothelium in HAE patients with severe disease may be more susceptible to damage or dysfunction, potentially predisposing them to bradykinin stimulation and resulting in more frequent or severe angioedema attacks.

LTP remains a significant clinical concern in hereditary angioedema HAE. Over the past decade, therapeutic advancements for HAE have been substantial. The global approval of selective plasma kallikrein inhibitors, such as lanadelumab and berotralstat, has enabled more patients to achieve rapid and sustained disease control while minimizing the side effects and inconvenience associated with plasma-derived C1-inhibitor (C1-INH) or androgen therapies [30, 31, 32, 33]. However, the high cost of monoclonal antibodies presents a considerable economic burden for patients and restricts their accessibility, particularly in developed countries. Hence, in terms of precision medicine paradigm, screening high-risk or potentially refractory patients to receive more active LTP is of clinical significance. Our findings identified the elevated VEGF level as an independent risk factor for severe and poorly controlled HAE, suggesting the potential predictive role of VEGF in stratifications of disease severity. Prospective follow-up studies are warranted to validate these findings. Before the advent of plasma kallikrein inhibitors, danazol was extensively utilized for LTP. Although the exact mechanism by which attenuated androgens exert their effects remains unclear, there is some evidence suggesting that low-dose danazol may enhance the barrier function of the vascular endothelium [34]. Additional research involving pairwise comparisons of circulating VEGF levels before and after danazol treatment in patients with HAE could provide further validation for this hypothesis. An animal study has demonstrated that administering an endothelial dysfunction blocker (CU06-1004) in SERPING1-deficient mice may mitigate vascular hyperpermeability by protecting the endothelial barrier function against bradykinin stimulation, suggesting that strategies aimed at protecting or stabilizing the vascular endothelium against bradykinin may offer an alternative prophylactic approach for HAE [35]. We anticipate the outcomes of further research translating these findings from animal models to human studies.

To the best of our knowledge, this study is the first to evaluate the correlation between circulating VEGF levels and HAE, as well as its disease status, in Chinese patients. Our findings indicate a compromised vascular function in HAE patients at baseline, contributing to the understanding of the mechanisms underlying the paroxysmal episodes of HAE. Additionally, these results highlighted potential utility of circulating VEGF levels in disease monitoring. However, this study has several limitations. Firstly, the single-center and single-ethnicity design restricts the generalizability of our results, and the retrospective nature of data collection might introduce recall bias. Thus, the predictive efficacy of VEGF in determining disease monitoring requires validation through future multi-center studies with prospective follow-up. Secondly, matching between compared groups was not performed. Although regression analyses were employed to address potential confounding effects, the presence of selection bias cannot be entirely ruled out. Thirdly, despite implementing restrictions on drug use prior to blood testing, the influence of drug interventions on the measurement of VEGF level may still exist. Fourthly, there is currently no standardized method for assessing HAE disease severity. In this study, disease severity was evaluated comprehensively based on published criteria and the expertise of our institution, which is not free of subjective judgment bias. Once a consensus is established, these analyses should be revised accordingly. In addition, the correlation between circulating VEGF levels and other significant patient-reported outcome measures, such as quality of life and disease activity, was not examined due to insufficient data. This limitation impedes a thorough assessment of the utility of VEGF levels in disease monitoring.

## Conclusion

During the attack-free period, circulating levels of VEGF remained elevated in patients with HAE, particularly among those exhibiting greater disease burden. Mechanistically, these findings suggest a persistent dysfunction of the vascular endothelium in HAE patients. Clinically, VEGF may serve as a predictive biomarker for risk stratification and disease monitoring in HAE. These findings warrant validation through further prospective cohort studies and animal research.

## Electronic supplementary material

Below is the link to the electronic supplementary material.

Supplementary Material 1

Supplementary Material 2

Supplementary Material 3

## Data Availability

The data that support the findings of this study are available from the corresponding author upon reasonable request.

## Abbreviations

HAE: Hereditary angioedema
VEGF: Vascular endothelium growth factor
C1: Complement 1
C1-INH: Complement 1-esterase inhibitor
C4: Complement 4
C1q: Complement 1q
STROBE-ME: Reporting of Observational Studies in Epidemiology-Molecular Epidemiology
BMI: Body mass index
IQR: Interquartile range
SD: Standard deviation
CI: Confidence interval
LTP: Long-term prophylaxis
GLMs: Generalized linear models
AECT: Angioedema control test
ROC: Receiver operating characteristic
AUC: Area under the curve
AIC: Akaike information criterion
IRR: Incidence rate ratios
RMSE: Root mean square error
VIF: Variance inflation factors
SE: Standard errors
