# Supplementary material for: Elevated level of circulating VEGF in Chinese patients with hereditary angioedema and its correlation with disease status
Source: Orphanet J Rare Dis. 2025 May 26;20:251. doi: 10.1186/s13023-025-03776-3 (PMC12105160; doi:10.1186/s13023-025-03776-3)
Supplement: Supplementary file 1 — Supplementary Material 1 [file 13023_2025_3776_MOESM1_ESM.docx]

**Table 1 Baseline demographic and disease characteristics of enrolled patients**

|  | HAE (n=74) | Non-hereditary AE (n=55) | P value |
| --- | --- | --- | --- |
| **Demographic characteristics** | | | |
| Age (years, median, IQR) | 41(33-54) | 43 (32-55) | 0.954 |
| Male (n, %) | 30 (40.5%) | 28 (50.9%) | 0.762 |
| BMI (kg/m^2^, median, IQR) | 21.7 (19.4-23.8) | 21.1 (19.6-23.0) | 0.675 |
| Non-allergic comorbidities (n, %) | 24 (32.4%) | 22 (40.0%) | 0.375 |
| Allergic comorbidity (n, %) | 6 (8.1%) | 24 (43.6%) | <0.001 |
| **Disease characteristics** | | | |
| Family history (n, %) | 63 (85.1%) | 5 (9.1%) | <0.001 |
| Age of onset (years, median, IQR) | 24.0 (16.8-29.3) | 39.0 (27.5-49.7) | <0.001 |
| Disease duration (years, median, IQR) | 18.0 (8.0-24.0) | 2.0 (0.5-5.0) | <0.001 |
| Hospitalization or emergency department visit (n, %) | 62 (83.8%) | 15 (27.3%) | <0.001 |
| History of laryngeal edema (n, %) | 45 (60.8%) | 14 (25.5%) | <0.001 |
| History of intense abdominal pain (n, %) | 51 (68.9%) | 9 (16.4%) | <0.001 |
| Concurrent urticaria (n, %) | 0 (0.0%) | 13 (23.6%) | <0.001 |
| **Drug interventions** | | | |
| Danazol (n, %) | 20 (27.0%) | 0 (0.0%) | <0.001 |
| Lanadelumab (n, %) | 5 (6.8%) | 0 (0.0%) |  |
| Second-generation antihistamines (n, %) | 0 (0.0%) | 24 (43.6%) |  |
| Glucocorticoid (n, %) | 0 (0.0%) | 2 (3.6%) |  |
| **Laboratory results** | | | |
| C1-INH/lower normal limit (median, IQR) | 0.24 (0.19-0.29) | 1.33 (1.19-1.52) | <0.001 |
| Complement 4 (mg/mL, median, IQR) | 0.053 (0.023-0.085) | 0.215 (0.178-0.269) | <0.001 |
| VEGF (ng/ml, median, IQR) | 112 (72-169) | 60 (39-80) | <0.001 |

HAE, hereditary angioedema; AE, angioedema; IQR, interquartile range; BMI, body mass index; C1-INH, complement 1-esterase inhibitor; VEGF, vascular endothelium growth factor; AECA, anti-endothelial cell antibody.


**Table 2 Univariate and multivariate analyses of the correlation between clinical variables and the disease severity of HAE**

| Variables | Univariate analysis | | | Multivariate analysis | | |
| --- | --- | --- | --- | --- | --- | --- |
|  | Odds ratio | 95% CI | P value | Odds ratio | 95% CI | P value |
| Age | 1.00 | 0.97-1.03 | 0.887 | 1.02 | 0.97-1.07 | 0.420 |
| Male | 1.31 | 0.51-3.23 | 0.573 | 1.79 | 0.41-7.88 | 0.441 |
| Comorbidity | 0.85 | 0.32-2.26 | 0.747 |  |  |  |
| Duration of disease course | 0.98 | 0.95-1.02 | 0.372 | - | - | - |
| Family history | 0.92 | 0.25-3.32 | 0.894 | - | - | - |
| Previous laryngeal edema | 5.81 | 2.08-16.29 | 0.001 | 25.04 | 3.62-173.45 | 0.001 |
| Long-term prophylaxis | 0.15 | 0.05-0.46 | 0.001 | 0.09 | 0.01-0.59 | 0.012 |
| C1-INH/lower normal limit | 0.23 | 0.030-1.74 | 0.153 | - | - | - |
| Complement 4/lower normal limit | 0.25 | 0.07-0.84 | 0.025 | 0.39 | 0.06-2.67 | 0.338 |
| VEGF level | 1.03 | 1.01-1.04 | <0.001 | 1.04 | 1.01-1.06 | 0.001 |

HAE, hereditary angioedema; CI, confidence interval; C1-INH, complement 1-esterase inhibitor; VEGF, vascular endothelium growth factor.
