# Supplementary material for: Elevated level of circulating VEGF in Chinese patients with hereditary angioedema and its correlation with disease status
Source: Orphanet J Rare Dis. 2025 May 26;20:251. doi: 10.1186/s13023-025-03776-3 (PMC12105160; doi:10.1186/s13023-025-03776-3)
Supplement: Supplementary file 2 — Supplementary Material 2 [file 13023_2025_3776_MOESM2_ESM.docx]

**Table S1 Strengthening the Reporting Observational studies in Epidemiology-Molecular Epidemiology (STROBE-ME) Reporting Recommendations: Extended from STROBE statement ^[1]^**

| **Item** | **Item number** | **STROBE Guidelines** | **Reporting Location** | **Extension for Molecular Epidemiology Studies (STROBE-ME)** | **Reporting Location** |
| --- | --- | --- | --- | --- | --- |
| **Title and abstract** | 1 | (a) Indicate the study’s design with a commonly used term in the title or the abstract | Page 2 Line 14 | **ME-1** State the use of specific biomarker(s) in the title and/or in the abstract if they contribute substantially to the findings | Page 1 Line 1, Page 2 Line 15 |
|  |  | (b) Provide in the abstract an informative and balanced summary of what was done and what was found | Page 2 |  |  |
| **Introduction** |  |  |  |  |  |
| Background rationale | 2 | Explain the scientific background and rationale for the investigation being reported | Page 4 Line 49-60 | **ME-2** Explain in the scientific background of the study how/why the specific biomarker(s) have been chosen, potentially among many others (e.g., others are studied but reported elsewhere, or not studied at all) | Page 4 Line 61-68 |
| Objectives | 3 | State specific objectives, including any pre-specified hypotheses | Page 4 Line 61-71 | **ME-3** *A priori* hypothesis: if one or more biomarkers are used as proxy measures, state the *a priori* hypothesis on the expected values of the biomarker(s) | Page 4 Line 68-71 |
| **Methods** |  |  |  |  |  |
| Study design | 4 | Present key elements of study design early in the paper | Page 4 Line 73 | **ME-4** Describe the special study designs for molecular epidemiology (in particular nested case/control and case/cohort) and how they were implemented | Page 4 Line 82 to Page 6 Line 125 |
| *Biological sample collection* |  |  |  | **ME-4.1** Report on the setting of the biological sample collection; amount of sample; nature of collecting procedures; participant conditions; time between sample collection and relevant clinical or physiological endpoints. | Page 6 Line 128-130 |
| *Biological sample storage* |  |  |  | **ME-4.2** Describe sample processing (centrifugation, timing, additives, etc). | Page 7 Line 131-132 |
| *Biological sample processing* |  |  |  | **ME-4.3** Describe sample storage until biomarker analysis (storage, thawing, manipulation, etc). | Page 7 Line 132-133 |
| *Biomarker biochemical characteristics* |  |  |  | **ME-4.4** Report the half-life of the biomarker, and chemical and physical characteristics (e.g., solubility). | NA |
| Setting | 5 | Describe the setting, locations, and relevant dates, including periods of recruitment, exposure, follow-up, and data collection | Page 5 Line 79-80 |  |  |
| Participants | 6 | (a) Cohort study—Give the eligibility criteria, and the sources and methods of selection of participants. Describe methods of  follow-up  Case-control study—Give the eligibility criteria, and the sources and methods of case ascertainment and control selection. Give  the rationale for the choice of cases and controls  Cross-sectional study—Give the eligibility criteria, and the sources and methods of selection of participants | Page 5 Line 81-96 | **ME-6** Report any habit, clinical conditions, physiological factor, or working or living condition that might affect the characteristics or concentrations of the biomarker | Page 5 Line 92-96 |
|  |  | (b) Cohort study—For matched studies, give matching criteria and number of exposed and unexposed  Case-control study—For matched studies, give matching criteria and the number of controls per case | NA |  |  |
| Variables | 7 | Clearly define all outcomes, exposures, predictors, potential confounders, and effect modifiers. Give diagnostic criteria, if applicable | Page 5 Line 98 to Page 7 Line 138 |  |  |
| Data source/measurement | 8 | For each variable of interest, give sources of data and details of methods of assessment (measurement).  Describe comparability of assessment methods if there is more than one group | Page 5 Line 98 to Page 7 Line 138 | **ME-8** Laboratory methods: report type of assay used, detection limit, quantity of biological sample used, outliers, timing in the assay procedures (when applicable) and calibration procedures or any standard used | Page 7 Line 134-138 |
| Bias | 9 | Describe any efforts to address potential sources of bias | Page 6 Line 124, Page 6 Line 128-129, Page 7 Line 137 |  |  |
| Study size | 10 | Explain how the study size was arrived at | Supplementary; Page 8 Line 173-175 |  |  |
| Quantitative variables | 11 | Explain how quantitative variables were handled in the analyses. If applicable, describe which groupings were chosen, and why | Page 6 Line 114-115 |  |  |
| Statistical methods | 12 | (a) Describe all statistical methods, including those used to control for confounding | Page 7 Line 140 to Page 8 Line 175 | **ME-12** Describe how biomarkers were introduced into statistical models | Page 7 Line 140 to Page 8 Line 171 |
|  |  | (b) Describe any methods used to examine subgroups and interactions | Page 7 Line 140 to Page 8 Line 175 |  |  |
|  |  | (c) Explain how missing data were addressed | Page 6 Line 112-113 |  |  |
|  |  | (d) Cohort study—If applicable, explain how loss to follow-up was addressed  Case-control study—If applicable, explain how matching of cases and controls was addressed  Cross-sectional study—If applicable, describe analytical methods taking account of sampling strategy | Supplementary; Page 8 Line 173-175 |  |  |
|  |  | (e) Describe any sensitivity analyses | NA |  |  |
| *Validity/reliability of measurement and internal/external validation* |  |  |  | **ME-12.1** Report on the validity and reliability of measurement of the biomarker(s) coming from the literature and any internal or external validation used in the study. | Page 7 Line 134-138 |
| **Results** |  |  |  |  |  |
| Participants | 13 | (a) Report the numbers of individuals at each stage of the study—e.g., numbers potentially eligible, examined for eligibility, con-  firmed eligible, included in the study, completing follow-up, and analysed | Figure 1 | **ME-13** Give reason for loss of biological samples at each stage | NA |
|  |  | (b) Give reasons for non-participation at each stage | Figure 1 |  |  |
|  |  | (c) Consider use of a flow diagram | Figure 1 |  |  |
| Descriptive data | 14 | (a) Give characteristics of study participants (e.g., demographic, clinical, social) and information on exposures and potential con-  founders | Page 8 Line 178-184; Table 1 |  |  |
|  |  | (b) Indicate the number of participants with missing data for each variable of interest | NA |  |  |
|  |  | (c) Cohort study—Summarise follow-up time (e.g., average and total amount) | NA |  |  |
| *Distribution of biomarker measurement* |  |  |  | **ME-14.1** Give the distribution of the biomarker measurement (including mean, median, range, and variance) | Page 9 Line 194 |
| Outcome data | 15 | Cohort study—Report numbers of outcome events or summary measures over time  Case-control study—Report numbers in each exposure category, or summary measures of exposure  Cross-sectional study—Report numbers of outcome events or summary measures | Page 8 Line 183 to Page 10 Line 240 |  |  |
| Main results | 16 | (a) Give unadjusted estimates and, if applicable, confounder-adjusted estimates and their precision (e.g., 95% confidence interval).  Make clear which confounders were adjusted for and why they were included | Page 9 Line 210 to Page 10 Line 240 |  |  |
|  |  | (b) Report category boundaries when continuous variables were categorized | Page 6 Line 114-115 |  |  |
|  |  | (c) If relevant, consider translating estimates of relative risk into absolute risk for a meaningful time period | NA |  |  |
| Other analyses | 17 | Report other analyses done—e.g., analyses of subgroups and interactions, and sensitivity analyses | Page 10 Line 241 to Page 11 Line 250 |  |  |
| **Discussion** |  |  |  |  |  |
| Key results | 18 | Summarise key results with reference to study objectives | Page 12 Line 252-263 |  |  |
| Limitations | 19 | Discuss limitations of the study, taking into account sources of potential bias or imprecision. Discuss both direction and magnitude  of any potential bias | Page 13 Line 313-327 | **ME-19** Describe main limitations in laboratory procedures | Page 13 Line 319-321 |
| Interpretation | 20 | Give a cautious overall interpretation of results considering objectives, limitations, multiplicity of analyses, results from similar studies, and other relevant evidence | Page 13 Line 310-313 | **ME-20** Give an interpretation of results in terms of *a-priori* biological plausibility | Page 11 Line 264 to Page 12 Line 283 |
| Generalisability | 21 | Discuss the generalisability (external validity) of the study results | Page 13 Line 314 |  |  |
| **Other information** |  |  |  |  |  |
| Funding | 22 | Give the source of funding and the role of the funders for the present study and, if applicable, for the original study on which the  present article is based | Page 17 Line 426-428 |  |  |
| Ethics |  |  | Page 17 Line 419-421 | **ME-22.1** Describe informed consent and approval from ethical committee(s). Specify whether samples were anonymous, anonymised or identifiable | Page 4 Line 73-75 |

**Table S2 Clinical characteristics between severe and non-severe HAE patients**

|  | HAE patients with severe disease status (n=39) | HAE patients with non-severe disease status (n=35) | P value |
| --- | --- | --- | --- |
| **Demographic characteristics** | | | |
| Age (years, median, IQR) | 41 (32-56) | 41(33-54) | 0.974 |
| Male (n, %) | 17 (43.6%) | 13 (37.1%) | 0.573 |
| BMI (kg/m^2^, median, IQR) | 21.5 (19.2-23.5) | 21.8 (19.7-24.0) | 0.295 |
| Comorbidities (n, %) | 12 (30.8%) | 12 (34.3%) | 0.747 |
| **Disease characteristics** | | | |
| Family history (n, %) | 33 (84.6%) | 30 (85.7%) | 0.894 |
| Family members died from HAE (n, %) | 9 (23.1%) | 6 (17.1%) | 0.526 |
| Age at onset of HAE symptoms (years, median, IQR) | 24 (17-27) | 24 (18-32) | 0.461 |
| Disease duration (years, age, median, IQR) | 15 (7-22) | 20 (8-26) | 0.368 |
| Type of HAE (n, %) | | | |
| Type I | 38 (97.4%) | 33 (94.3%) | 0.600 |
| Type II | 1 (2.6%) | 2 (5.7) |  |
| Hospitalization or emergency department visit (n, %) | 36 (92.3%) | 26 (74.3%) | 0.036 |
| History of laryngeal edema (n, %) | 31 (79.5%) | 14 (40.0%) | 0.001 |
| History of intense abdominal pain (n, %) | 29 (74.4%) | 22 (62.9%) | 0.167 |
| **Disease control status over the past six months** (n, %) | | | |
| Long-term prophylaxis (n, %) | | | |
| Lanadelumab | 0 (0.0%) | 5 (14.3%) | <0.001 |
| Danazol | 6 (15.4%) | 14 (40.0%) |  |
| No | 33 (84.6%) | 16 (45.7%) |  |
| Number of angioedema attacks over the past six months (n, %) | | | |
| 0 | 0 (0.0%) | 14 (40.0%) | <0.001 |
| 1-5 | 19 (48.7%) | 21(60.0%) |  |
| ≥6 | 20 (51.3%) | 0 (0.0%) |  |
| Laryngeal edema (n, %) | 17 (43.6%) | 0 (0.0%) | <0.001 |
| Intense abdominal pain (n, %) | 10 (25.6%) | 4 (11.4%) | 0.119 |
| Hospitalization or emergency department visit (n, %) | 19 (48.7%) | 3 (8.6%) | <0.001 |
| **Laboratory results** | | | |
| C1-INH/lower normal limit (%, median, IQR) | 0.29 (0.19-0.38) | 0.33 (0.25-0.48) | 0.082 |
| Complement 4 (mg/mL, median, IQR) | 0.043 (0.020-0.070) | 0.074 (0.036-0.102) | 0.020 |
| VEGF (ng/ml, median, IQR) | 165 (112-203) | 80 (61-112) | <0.001 |

HAE, hereditary angioedema; IQR, interquartile range; BMI, body mass index; C1-INH, complement 1-esterase inhibitor; VEGF, vascular endothelium growth factor

**Sample size estimation**

The estimation of sample size was performed utilizing G*Power software (Version 3.1.9.7, Germany). The δ means and pooled standard deviation (SD) values were derived from the data published by Loffredo et al.[2] and our preliminary analysis. The effect size (d) was calculated to range between 0.60 and 0.80 (δ means/pooled SD), with the more conservative value of 0.60 being selected as the effect size. The t-test family was chosen, and the statistical test was specified as "Means: Wilcoxon-Mann-Whitney test (two groups)." The significance level (α) was set at 0.05 for a two-tailed test, and the power (1-β) was set at 0.90. The results indicated that the required sample size for each group was approximately 65.

**Post-hoc power calculation**

We calculated the post-hoc power by the differences in VEGF levels between HAE and AE patients, which is our major outcome. We utilized G*Power software (Version 3.1.9.7, Germany) for the power calculation. We selected the t-test family and specified the statistical test as "Means: Wilcoxon-Mann-Whitney test (two groups)." The mean VEGF levels of two groups were 129.2 and 69.2 ng/ml, with a pooled standard deviation of 59.8. Consequently, the effect size (d) was calculated to be 1.14 (δ means/pooled SD). By inputting the effect size, sample size of each group and setting the significance level (α) at 0.05, the calculated power (1-β) exceeded 0.999, indicating a strong post-hoc power.

**References**

1. Gallo, V., et al., *STrengthening the Reporting of OBservational studies in Epidemiology - Molecular Epidemiology (STROBE-ME): an extension of the STROBE statement.* Eur J Clin Invest, 2012. **42**(1): p. 1-16.

2. Loffredo, S., et al., *Elevated plasma levels of vascular permeability factors in C1 inhibitor-deficient hereditary angioedema.* Allergy, 2016. **71**(7): p. 989-96.
