# Supplementary figures and images for: Elevated level of circulating VEGF in Chinese patients with hereditary angioedema and its correlation with disease status
Source: Orphanet J Rare Dis. 2025 May 26;20:251. doi: 10.1186/s13023-025-03776-3 (PMC12105160; doi:10.1186/s13023-025-03776-3)

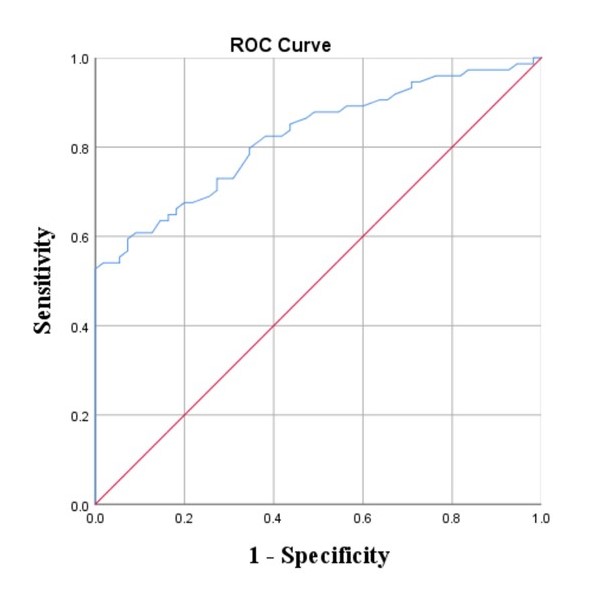

Supplement: Supplementary file 3 — Supplementary Material 3 [file 13023_2025_3776_MOESM3_ESM.jpg]
